# Monitoring Aflatoxin M1 in Milk From Selected Iranian Provinces Using Different Assays: Seasonal Trends and Processing Effects

**DOI:** 10.1002/fsn3.71027

**Published:** 2025-09-26

**Authors:** Hamid Reza Fathali Beygi, Mojtaba Jafari, Zohreh Ashrafi, Bahram Hassani, Razie Razavi

**Affiliations:** ^1^ Department of Food Science and Technology, Faculty of Science and Technology Islamic Azad University, North Tehran Branch Tehran Iran; ^2^ Department of Food Science and Technology, Faculty of Industrial and Mechanical Engineering Islamic Azad University, Qazvin Branch Qazvin Iran; ^3^ Department of Biology, Faculty of Science Payame Noor University, East Tehran Branch Tehran Iran; ^4^ Department of Food Industry Ferdowsi University of Mashhad Mashhad Iran; ^5^ Department of Food Science and Technology Sari Agricultural Sciences and Natural Resources University (SANRU) Sari Iran

**Keywords:** aflatoxin M1, food contamination, pasteurization, raw milk, risk assessment, spray drying

## Abstract

Accurate and rapid determination of aflatoxin M1 (AFM1) is essential for ensuring the safety of milk and dairy products. This study is the first to integrate seasonal analysis, processing effects, multi‐method comparison, and MOE‐based risk assessment of AFM1 in Iranian milk, providing comprehensive insights for food safety monitoring. A total of 227 raw milk samples from 12 provinces were analyzed across different seasons using immunochromatography, ELISA, and HPLC. The prevalence of AFM1 exceeding the legal limit of 0.10 μg/kg, as set by the Institute of Standards and Industrial Research of Iran (ISIRI), was 9.7% in spring, 13.3% in summer, 3.7% in autumn, and 26.4% in winter. The highest average AFM1 concentrations were recorded in winter and summer, while the lowest occurred in spring and autumn. The study also evaluated the effects of pasteurization and spray‐drying on AFM1 levels in skim milk and skim milk powder, with HPLC analysis showing no significant changes (*p* ≥ 0.05) post‐processing. Risk assessment calculated the estimated daily intake (EDI) and hazard index (HI) for liver cancer, with EDI in winter reaching 62.58 ng/kg body weight and HI at 1.06, based on a tolerable daily intake (TDI) of 0.2 ng/kg bw/day derived from TD50 with a safety factor of 50,000. These results highlight the need for continuous monitoring and regulation of AFM1 to safeguard public health. Among the methods tested, immunochromatography demonstrated clear advantages over ELISA and HPLC, making it an efficient and practical tool for rapid preliminary detection of AFM1 in milk.

## Graphical abstract

Rapid measurement of aflatoxin in milk is very important as a common toxin. It affects the acceptance of milk cargo. In this study, 227 milk samples were collected from different provinces of Iran, and their aflatoxin levels were measured using HPLC, ELISA, and immunochromatographic techniques. The results showed that the immunochromatographic techniques can be used as a quick method to determine the content of aflatoxin, which can help us to process milk properly based on the content of aflatoxin. Seasonal variation in AFM1 concentrations of raw milk samples.

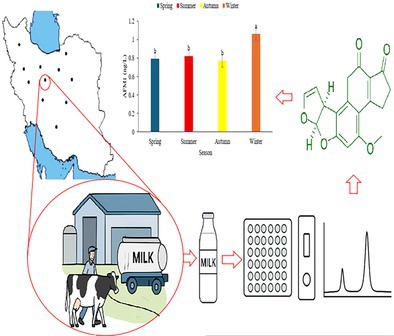

## 1 Introduction

Milk is a nutritionally rich food containing essential macro‐ and micronutrients, though its composition varies with factors such as breed, diet, and season. However, it may also serve as a vehicle for contaminants like AFM1, a mycotoxin of concern globally over the several decades (Ghaffarian‐Bahraman et al. 2023; Iqbal et al. 2015; Kenari and Razavi 2021; Nadira et al. 2017; Zebib et al. 2023). According to the European Food Safety Authority, aflatoxins, including AFM1, are considered genotoxic and carcinogenic, posing a significant health risk particularly for infants and young children due to their higher exposure levels relative to body weight (Chain et al. 2020). Over the last decade or two, the issue of AFM1 contamination has become particularly significant, posing a major challenge for public health, especially in low‐ and middle‐income countries, where regulatory control and monitoring systems may be less stringent (Ghaffarian‐Bahraman et al. 2023; Iqbal et al. 2015). The primary cause of aflatoxin contamination in milk samples is the consumption of feed contaminated with mycotoxins by lactating animals. Depending on the type of mycotoxin, these contaminated feedstuffs may lead to the presence of metabolized substances or their derivatives (modified mycotoxins) in raw milk samples (Freire and Sant'Ana 2018).

The contamination of livestock feed with mycotoxins is a global concern within the animal products industry, posing significant threats to both animal and human health. Mycotoxins can exert inhibitory, mutagenic, carcinogenic, and immunosuppressive effects (Iqbal et al. 2015). Given the detrimental impacts of aflatoxins, which include toxicity, carcinogenicity, mutagenicity, and immunosuppression, the widespread occurrence of various aflatoxins in food and feed presents considerable risks to human and animal health (Thakur et al. 2022).

Aflatoxins are toxic secondary metabolites predominantly synthesized by the fungi *Aspergillus flavus* and *Aspergillus parasiticus*, with occasional contributions from strains such as *Aspergillus tamarii*, *Aspergillus nomius*, and *Aspergillus pseudotamarii*. These mycotoxins are produced in a variety of agricultural products under specific conditions of temperature and humidity during both harvesting and post‐harvest processing (Ramadan and Al‐Ameri 2022).

The prevalence of aflatoxins in crops is largely determined by climatic conditions. Arid and high‐temperature environments favor the proliferation of storage fungi, thereby enhancing the synthesis of aflatoxins (Panara et al. 2022; Stefanovic et al. 2015). AFM1, a hepatocarcinogenic metabolite formed from aflatoxin B1 (AFB1) through hydroxylation in the liver, can lead to DNA damage and chromosomal aberrations, contingent upon exposure levels. It is excreted into the milk of lactating animals ingesting AFB1‐contaminated feed via hepatic microsomal cytochrome P450 activity (Cao et al. 2022; Michlig et al. 2016). AFM1, classified as a group 1 carcinogen by the World Health Organization (WHO), and therefore, no Tolerable Daily Intake (TDI) should be established for this substance. The Margin of Exposure (MOE) approach is more appropriate for assessing the risk of carcinogenic substances, and this method was applied in our risk assessment calculations. AFM1 exhibits a carcinogenicity level of 2%–10% relative to AFB1 (Schincaglia et al. 2023; Stefanovic et al. 2015).

Over 300 aflatoxin types have been identified. The research shows a direct correlation between AFM1 concentration in milk and AFB1 levels in animal feed, with 2%–6% of ingested AFB1 converting to AFM1 in milk. AFM1 can be detected in milk 12–24 h post‐ingestion of contaminated feed and diminishes to undetectable levels within 72 h after discontinuation of exposure to contaminated feed (Costamagna et al. 2024; Fallah et al. 2016; Flores‐Flores et al. 2015; Ketney et al. 2017).

To safeguard consumers, especially infants and children, various countries have established maximum residue limits (MRLs) for AFM1 in milk and milk products. These MRLs vary by country, reflecting differences in hygienic standards, economic considerations, and risk assessments. The European Commission has set an MRL of 0.050 μg/L, while the US FDA and most Asian countries allow up to 0.50 μg/L. The Institute of Standards and Industrial Research of Iran (ISIRI) has approved a stricter limit of 0.100 μg/L, which is more conservative than the limits set by the US and European Commission. Studies have indicated that 0.05 μg/kg of AFM1 in milk can be associated with an intake of 40 μg/kg of AFB1 per day per dairy cow (Zentai et al. 2023). Ongoing risk assessments are essential for the surveillance and prevention of potential AFM1 contamination, necessitating the use of rapid, cost‐effective, and high‐capacity screening techniques (Conteçotto et al. 2021; Movassaghghazani and Shabansalmani 2024; Neshovska et al. 2020; Venâncio et al. 2019).

High‐Performance Liquid Chromatography (HPLC) and Thin Layer Chromatography (TLC) are critical methods employed for detecting AFM1 in various foodstuffs. Enzyme‐linked immunosorbent Assay (ELISA), HPLC, and TLC are common methods for the quantification of AFM1 (Esam et al. 2022). Furthermore, the study explores the impact of seasonal and thermal variations on AFM1 levels during the conversion of high‐fat milk to skim milk powder. Additionally, the potential link between AFM1 exposure and liver cancer risk is examined through the calculation of EDI, providing a nuanced understanding of AFM1's health implications (Campagnollo et al. 2016; Ketney et al. 2017).

Given the precise quantification capabilities of HPLC, it is widely adopted as the standard reference method for aflatoxin measurement in numerous countries, including Iran. Similarly, the ELISA method, known for its relative accuracy, is considered a suitable alternative. However, these methods face significant limitations such as being time‐intensive, costly, non‐portable, complex, and requiring skilled personnel, which restricts their applicability across various settings and conditions. In contrast, immune chromatography offers a rapid, user‐friendly, cost‐effective, and reliable alternative for aflatoxin measurement. To the best of our knowledge, there has been no prior comprehensive study on this topic. The findings of this study will provide a valuable database for regulatory authorities, assist food industry professionals, increase consumer awareness, and support the management of AFM1 contamination in raw milk and dairy products in Iran.

Several studies have reported the presence of AFM1 in milk across different regions (Behtarin and Movassaghghazani 2024; Mohammedi‐Ameur et al. 2020), with contamination levels often influenced by factors such as seasonal variations (Akbar et al. 2019; Bilandžić et al. 2022), feed storage conditions (Admasu et al. 2021; Patyal et al. 2020), and regional climatic patterns (Daou et al. 2022). For instance, research from Iran, Turkey, Pakistan, and India has demonstrated higher AFM1 levels during colder months due to increased consumption of contaminated stored feed (Akbar et al. 2019; Rahimzadeh Barzoki et al. 2023; Yalçin et al. 2022). In Europe, particularly in Mediterranean countries, seasonal patterns also appear to affect contamination levels (Malissiova et al. 2024).

While many studies have utilized ELISA or HPLC for AFM1 detection, comparative data on the performance of various detection methods in field settings remain limited (Maggira et al. 2021). Furthermore, despite growing awareness, comprehensive data on seasonal trends in AFM1 contamination within Iran remain scarce, especially in relation to different milk processing techniques. The inclusion of both seasonal trend analysis and the assessment of pasteurization effects in this study allows for a more comprehensive understanding of how environmental factors and processing conditions influence AFM1 contamination levels in milk. Seasonal variations in AFM1 levels can be influenced by changes in feed quality and storage conditions, while pasteurization represents a critical processing step that may impact the concentration of this mycotoxin. The objectives of this study were: (i) to monitor AFM1 levels in raw milk collected from 12 provinces of Iran across different seasons; (ii) to evaluate the impact of milk processing, including pasteurization and spray drying, on AFM1 concentrations; (iii) to compare the performance of three analytical methods—immunochromatography, ELISA, and HPLC—for AFM1 detection; and (iv) to apply the Margin of Exposure (MOE) approach for risk assessment. These objectives collectively aim to generate novel insights into the dynamics of AFM1 contamination in Iran, inform regulatory authorities, and support evidence‐based strategies for improving dairy safety. By addressing these aims, the study seeks to provide evidence‐based insights that support improved food safety monitoring and regulatory policies. While the study builds on existing research frameworks, it introduces new insights by comparing multiple analytical methods for AFM1 detection in raw milk, evaluating the impact of milk processing techniques on AFM1 concentrations, and providing a comprehensive seasonal analysis. These contributions are crucial for advancing food safety practices and regulatory standards in Iran. This study provides a 1‐year seasonal snapshot of AFM1 contamination in the Iranian milk supply chain feeding into Tehran. While these data reveal important seasonal patterns, they should not be interpreted as long‐term climatic trends. Multi‐year monitoring is recommended to confirm and extend these findings.

## 2 Materials and Methods

### 2.1 Materials

ELISA Kit (RIDASCREEN) was purchased from R‐Biopharm (Darmstadt, Germany). Standard AFM1 solution and the AFLAPREP M safety column were purchased from R‐Biopharm Rhône Ltd. (Glasgow, Scotland). All reagents including acetonitrile, methanol, PBS buffer (pH = 7.4), chromogen, and sulfuric acid (H_2_SO_4_) were of HPLC grade and obtained from Mark (Darmstadt, Germany). Afla‐sensor Quanti KIT041 was purchased from Unisensor (Brussels, Belgium).

### 2.2 Methods

#### 2.2.1 Sample Collection

A total of 227 milk samples were randomly collected from bulk milk tanks at industrial centers 48 industrial milk collection centers and major milk producers across 12 provinces of Iran, namely Tehran, Esfahan, Semnan, Qazvin, Hamadan, Qom, Zanjan, Kerman, Markazi, Chaharmahal and Bakhtiari, Mazandaran, and East Azerbaijan, between spring 2019 and winter 2020. Provinces were selected because they directly or indirectly supply milk to Tehran. The sampling strategy was designed to represent the supply chain to the capital rather than the entire country. To minimize variability due to feeding practices, efforts were made to collect from farms with relatively uniform feeding conditions. In this study, a higher number of milk samples was collected during the winter season due to the commonly reported increased levels of aflatoxin M1 in milk during colder months, which is attributed to greater reliance on stored feed for dairy animals and favorable conditions for fungal growth. To minimize variability due to feeding practices, efforts were made to collect milk samples from farms and suppliers with relatively uniform feeding conditions. All centers supplied raw milk to Tehran, ensuring regional representativeness of the supply chain. Each sample comprised a minimum of 1000 mL of fresh raw milk, directly drawn from bulk milk tanks using sterile equipment. Samples were carefully labeled, immediately placed in sterile plastic containers, and transported to the laboratory in iceboxes (4°C) under cold chain conditions to maintain sample integrity and prevent aflatoxin degradation. Upon arrival, samples were stored in the dark at 4°C until testing. For analysis, 2 mL of each sample was used, while the remaining portion was frozen at −18°C for future assessments or confirmatory tests. To ensure accuracy in method development and instrument calibration, raw milk from a selected industrial center, verified as AFM1‐free by HPLC, was used as a negative control (Makau et al. 2016). Additionally, six samples were randomly selected and converted into skim milk powder at a processing facility in Qom province to assess the potential effect of drying and processing on AFM1 levels.

#### 2.2.2 Sample Preparation

**Raw Milk:** Samples were defatted by centrifugation (3500 rpm, 10°C, 10 min), and the top fat layer was removed using a vacuum pump.

**Skim Milk Powder:** Raw milk was first separated (Westfalia, Germany), pasteurized at 72°C for 15 s, and concentrated to Brix 50 (RV8, IKA, Germany). Spray drying was performed using a GEA Niro Spray Dryer (190°C). The concentration factor due to moisture loss was estimated as 7.5 (Hasanoğlu and Gül 2016).

#### 2.2.3 Instrumental Analysis

##### 2.2.3.1 Immunochromatographic Assay

A quantitative afla‐sensor strip test was used with a detection range of 20–200 ppt. Samples were incubated at 40°C, strips inserted after 3 min, and removed after 7 min. Strips were then read in a sensor device, and AFM1 concentration was determined via calibration curve (Talevski 2018).

##### 2.2.3.2 Elisa

ELISA kits (96‐well) were used following manufacturer protocols. Standard curves (50–4000 ng/L) were prepared using diluted AFM1 stock solution in acetonitrile. Samples and standards were loaded into wells, washed, incubated with enzyme‐conjugate, and treated with substrate and chromogen. After 30 min dark incubation, the reaction was stopped with H_2_SO_4_, and absorbance was read at 450 nm using an ELISA reader (Makau et al. 2016).

##### 2.2.3.3 HPLC

HPLC analysis was conducted using Waters 2695 (USA) with Discovery C18 columns and FLD (excitation/emission: 365/435 nm). Sample preparation included heating (35°C), centrifugation (4000 rpm, 12 min), fat removal, and immunoaffinity column cleanup. Elution was done with methanol‐acetonitrile (40:60, v/v), followed by nitrogen drying at 40°C. Samples were analyzed at different AFM1 concentrations (0.25 to 1 ng/L) (Maggira et al. 2021).

#### 2.2.4 Method Validation

All raw milk samples were tested by all three methods (immunochromatography, ELISA, and HPLC). For immunochromatography, negative and positive controls were included in every run. ELISA was validated for intra‐ and inter‐assay variability. HPLC validation included linearity, recovery (79%–94%), repeatability relative standard deviation (RSD) (~10%), and limit of detection (LOD)/limit of quantification (LOQ) determination. Offset sensor accuracy was validated using spiked samples (0–100 ng/L) across multiple sessions with different reagent batches and operators (5 replicates per level).

#### 2.2.5 Risk Assessment

##### 2.2.5.1 Estimation of Daily AFM1 Consumption

EDI of average aflatoxin weight is calculated for the adult population (age 18 and 27 and mean body weight = 70 kg) based on Equation (1) for daily consumption of AFM1 via milk consumption based on the recommended method of Cano‐Sancho and coworkers (Kaur et al. 2021). It is important to note that the carcinogenic potency of AFM1 is approximately 10% of AFB1, and this has been considered in our risk assessment calculations.

(1) $$\begin{array}{c} \mathrm{EDI}\left(\mathrm{\mu g}/\mathrm{kg}/\mathrm{day}\right)=\frac{\text{Daily intake level of milk}\;\left(\mathrm{kg}/\mathrm{day}\right)\times \text{AflatoxinM}1\;\text{level in milk}\left(\mathrm{\mu g}/\mathrm{kg}\right)}{\text{Average individual weight}\left(\mathrm{kg}\right)} \end{array}$$

##### 2.2.5.2 Risk Characterization

To characterize the health risk associated with AFM1 intake from milk, the Margin of Exposure (MOE) approach was applied, following the methodology recommended by the European Food Safety Authority (EFSA Panel on Contaminants in the Food Chain (CONTAM) et al. 2020). The MOE is defined as the ratio between a benchmark dose lower confidence limit (BMDL10) and the estimated daily intake (EDI) of the compound:

(2) $$\mathrm{MOE}=\frac{10\text{BMDL}}{\mathrm{EDI}}$$

In this equation, the BMDL10 for AFM1 was assumed to be 0.4 μg/kg body weight/day, based on its known carcinogenic potency. MOE values less than 10,000 were interpreted as indicating a potential public health concern, in line with EFSA's risk assessment criteria.

##### 2.2.5.3 Justification for Avoiding Hazard Index Approach

Due to the genotoxic and carcinogenic properties of aflatoxin M1, and in line with EFSA recommendations (EFSA Panel on Contaminants in the Food Chain (CONTAM) et al. 2020), the calculation of a Tolerable Daily Intake (TDI) and the use of the Hazard Index (HI) are considered inappropriate. Therefore, only the Margin of Exposure (MOE) approach was used in this study to assess cancer risk from AFM1 exposure.

#### 2.2.6 Verification and Approval of the Method

The accuracy and recovery of the evaluated offset sensor method were tested by analyzing raw milk samples treated with varying concentrations of AFM1 (0, 25, 50, 75, and 100 ng/L). Each concentration level was tested in five replicates. The analyses were performed across three sessions using similar tools and equipment but different reagent batches and operators (Khilosia 2011). Additional details regarding method validation procedures are provided in the Supporting Information.

### 2.3 Statistical Analysis

All samples were analyzed using immunochromatography, ELISA, and HPLC. Validation included recovery, RSD, LOD/LOQ, and intra−/inter‐assay variation. All experiments were conducted in five replicates, and data are presented as mean ± standard deviation (SD). Prior to statistical analysis, the Shapiro–Wilk test was used to assess the normality of the data distribution, and Levene's test was applied to verify the homogeneity of variances. To compare AFM1 concentrations across different seasons and provinces, one‐way analysis of variance (ANOVA) was employed. When significant differences were detected (*p* ≤ 0.05), Duncan's multiple range test was used for post hoc comparisons. To assess the effects of milk processing (raw vs. skim milk powder), independent samples *t*‐tests were conducted. For method comparison among immunochromatography, ELISA, and HPLC, Pearson correlation coefficients and Bland–Altman plots were used to evaluate agreement and consistency between analytical methods. All statistical analyses were carried out using IBM SPSS Statistics version 26.0 (IBM Corp., Armonk, NY, USA), and a significance level of *p* ≤ 0.05 was considered statistically significant. Prior to analysis, datasets were cleaned to eliminate outliers and incomplete records.

## 3 Results and Discussion

Recent research has investigated the occurrence and levels of AFM1 in various regions of Iran, revealing the unfortunate presence of this toxin in many milk and dairy products. The presence of AFM1 in 227 raw milk samples collected throughout all seasons from 2019 to 2020 was measured using the immunochromatography method. Table S2 shows the AFM1 concentration across dairy farms and sampling seasons. The analysis of AFM1 levels in raw milk across different seasons (Table 1) reveals statistically significant seasonal fluctuations (*p* ≤ 0.05). The highest mean AFM1 level was observed during summer (49.16 ± 6.3 ng/L), while the lowest was in autumn (46.35 ± 8.1 ng/L). Despite this, winter exhibited the most alarming profile in terms of public health, with 26.4% of samples exceeding the Iranian standard (100 ng/L) and 45.9% surpassing the EU limit (50 ng/L).

**TABLE 1:** Occurrence of AFM1 (ng/L) in raw milk measured by immunochromatography method.

Season	Number of samples	AFM1 content	The incidence rate higher than the range of			
			European standard	Iranian standard	Minimum	Maximum
Spring	41	47.54 ± 0.8^b^	13.41 (31.7%)^d^	4.41 (9.7%)^c^	20.0	150.0
Summer	45	49.16 ± 0.3^a^	17.45 (37.7%)^c^	6.54 (13.3%)^b^	20.0	150.0
Autumn	54	46.35 ± 0.1^c^	23.54 (42.5%)^b^	2.54 (3.7%)^d^	20.0	150.0
Winter	87	47.1 ± 0.4^b^	40.87 (45.9%)^a^	23.87 (26.4%)^a^	20.0	150.0

*Note:* Results reported as MD ± SE. Different lower‐case superscript letters exhibited statistically significant differences (*p* ≤ 0.05).

These seasonal fluctuations can be attributed to several interconnected factors. Table S1 shows the relative humidity and temperature of provinces studied in the present study. Firstly, climatic conditions play a critical role in the production of aflatoxins (Venâncio et al. 2019). In warmer months like summer, higher temperatures and humidity create favorable environments for the growth of *Aspergillus* species, which produce aflatoxin B1 in feed (Van der Fels‐Klerx et al. 2019). However, despite this, the peak contamination was observed in winter, suggesting that feed storage practices may have a greater impact during colder months (Xu et al. 2021). In winter, livestock are typically fed stored feed for longer durations due to limited fresh forage availability (Özbey et al. 2023).

**FIGURE 1:**
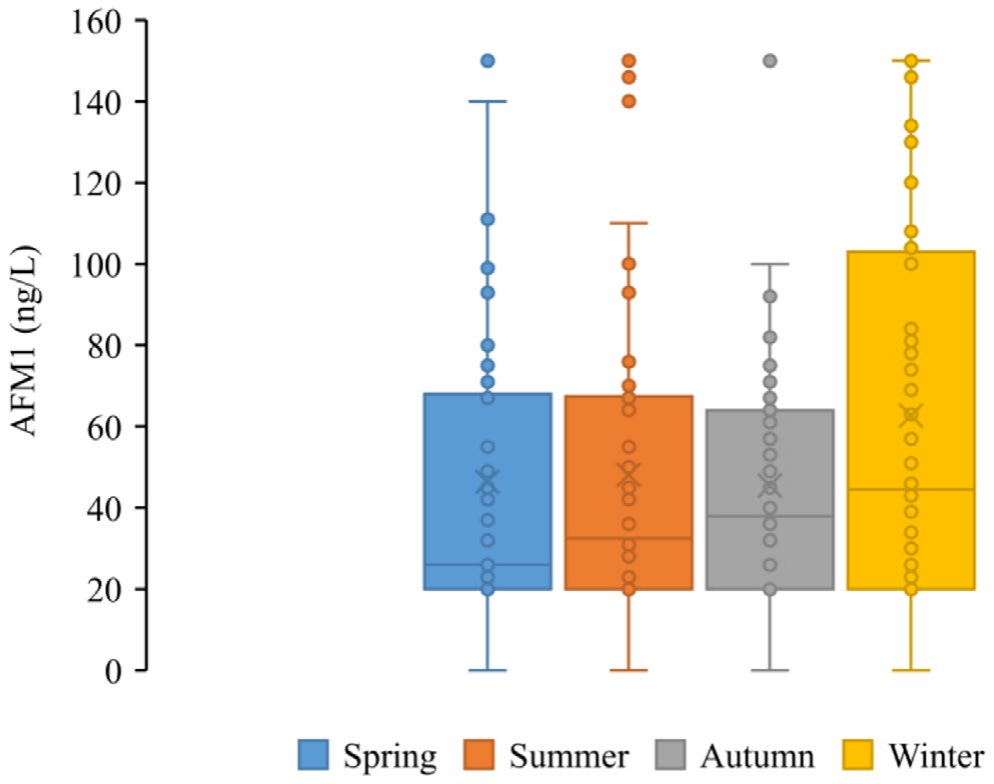
Seasonal variation in AFM1 concentrations of raw milk samples (box plot showing median, interquartile ranges, and outliers).

**FIGURE 2:**
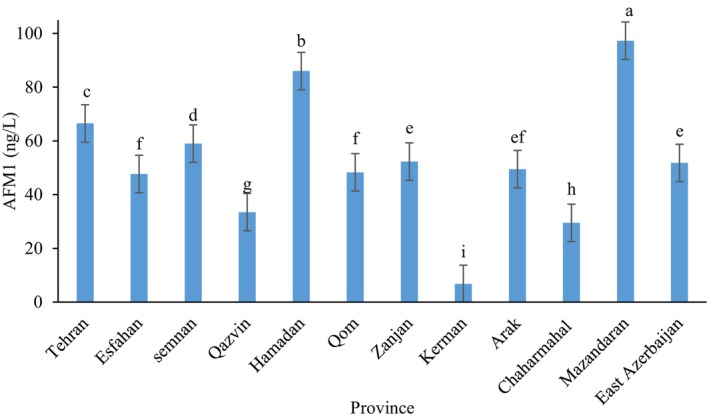
Geographical variation of AFM1 concentrations across 12 provinces supplying raw milk to Tehran during 2019 and 2020 determined by immunochromatography method (mean values ± SD). Different lowercase letters indicate the statistically significant difference (*p* ≤ 0.05) between AFM1 content of different provinces.

Boxplot analysis revealed clear seasonal variations in AFM1 concentrations in milk samples (Figure 1). The median and interquartile ranges were noticeably higher in winter, indicating elevated contamination levels during this period. Moreover, a greater number of outliers and extreme values were observed in winter, suggesting sporadic but significant AFM1 spikes, likely due to higher reliance on stored or contaminated feed. In contrast, summer and spring showed lower median values and narrower interquartile ranges, reflecting relatively stable and reduced AFM1 contamination. These findings support the hypothesis that seasonal factors, particularly those related to feeding practices and storage conditions, significantly influence AFM1 levels in dairy products. If storage conditions are suboptimal—such as high moisture levels, poor ventilation, or prolonged storage—aflatoxin levels in feed can increase substantially. Moreover, the quality and type of animal feed varies seasonally (Akbar et al. 2019). In summer and spring, animals may have more access to fresh pasture, reducing reliance on potentially contaminated stored feed (Polano et al. 2024). In contrast, the increased use of concentrated and stored feeds in winter could contribute to the higher AFM1 levels observed. Additionally, cold weather might influence the metabolic rate of livestock, potentially affecting how aflatoxins are metabolized and excreted into milk as AFM1 (Pate 2019; Wang et al. 2023). Furthermore, management practices in dairy farms, including feed rotation, storage infrastructure, and monitoring systems, might differ between seasons and farms, influencing the final contamination levels. Collectively, these findings highlight the importance of implementing season‐specific strategies for controlling aflatoxin contamination, particularly improving storage conditions and feed quality monitoring during the winter months (Figure 2).

The findings of this study reveal important regulatory implications regarding AFM1 contamination in milk, particularly when compared with international safety standards. In Iran, the national maximum residue limit (MRL) for AFM1 in milk is set at 100 ng/L (ISIRI 2013), whereas the European Union (EU) applies a stricter limit of 50 ng/L (EU 2023), and the United States Food and Drug Administration (FDA) permits levels up to 500 ng/L (FDA 2005), primarily due to differences in dietary patterns and risk management policies. Moreover, when assessed against the more rigorous EU threshold, the incidence of non‐compliance was notably higher across all seasons indicating a widespread public health concern. These findings suggest that while a portion of milk samples may meet the national standard, a substantial number would be deemed non‐compliant under EU regulations, which are often adopted as international benchmarks due to their precautionary approach. The elevated prevalence of AFM1 contamination—particularly during winter (Xu et al. 2021) months—raises critical questions about the adequacy of current national regulatory limits, the effectiveness of monitoring and enforcement, and the resilience of feed supply chains and storage practices under seasonal stress (Adelusi et al. 2023; Negash 2018). From a policy perspective, there may be a need to re‐evaluate existing national standards, enhance routine surveillance programs, and invest in preventive measures, such as improved feed quality control and farmer education, to ensure alignment with international safety goals and protect consumer health, especially for vulnerable groups such as infants and young children. Aligning national limits with global standards or implementing dual‐threshold systems may be considered as part of an integrated risk management strategy. Overall, the study underscores the importance of regulatory vigilance and international harmonization in managing the risk of AFM1 exposure through milk products.

Figure 2 presented the average levels (ng/L) of AFM1 in raw milk of 12 different provinces in Iran during 2019 and 2020 determined by the immunochromatography method. The levels of AFM1 in raw milk from various livestock farms, which were either directly or indirectly sent to Tehran province, generally were in the range of the Iranian standard limits on average. However, there was one exception where a particular livestock farm exhibited average AFM1 levels that surpassed the legal threshold set by ISIRI. The mean AFM1 (Aflatoxin M1) concentrations in raw milk samples collected from various provinces in Iran showed significant variation among provinces (*p* ≤ 0.05). The highest concentration was observed in Mazandaran province (95.31 ng/L), followed by Hamadan (84.3 ng/L), both of which exceeded the national maximum permissible limit (100 ng/L) in some samples. In contrast, Kerman showed the lowest contamination level (14.78 ng/L), indicating significantly safer milk quality in that region. Provinces such as Tehran, Semnan, and Zanjan exhibited moderate AFM1 levels ranging from 55 to 70 ng/L, while Chaharmahal and Qazvin showed relatively lower concentrations, around 30–40 ng/L. Overall, the data highlight clear geographical differences in AFM1 contamination, which could be attributed to varying factors such as climate conditions, feed storage practices, and regional dairy management. In research conducted by Rahimzadeh Barzoki et al. (2023), a total of 180 raw cow's milk samples were obtained from various retail dairy outlets in Gorgan, with 45 samples collected in each season. The concentration of AFM1 in these samples was assessed using the ELISA technique. AFM1 was detected in 139 samples, accounting for 72.2% of the total, with levels ranging between 3.5 and 357 ng/L. None of the samples exceeded the FDA's permissible threshold of 500 ng/L for AFM1. However, 41 samples (22.7%) surpassed the European Union's stricter limit of 50 ng/L, and 26 samples (14.4%) exceeded Iran's national maximum limit of 100 ng/L. Seasonal variations were notable, with the lowest contamination rates found in summer (32 samples, 71.1%) and the highest in winter (38 samples, 84.4%). Notably, the AFM1 levels in raw milk during winter were even higher than those observed in this study (Rahimzadeh Barzoki et al. 2023). Shabansalmani and Movassaghghazani (2023) conducted an analysis of AFM1 contamination in various types of milk—raw, pasteurized, and UHT—collected from Tehran. A total of 75 samples were examined, and the results revealed that AFM1 levels in all samples exceeded the maximum allowable limits set by both the European Union and Iran. The study also highlighted that children in Tehran faced the highest dietary intake and hazard index related to AFM1, with contamination levels peaking during the autumn season (Shabansalmani and Movassaghghazani 2023).

Previous research has highlighted the influence of environmental and climatic conditions, livestock health, weather patterns, and rainfall on the levels of AFM1 in milk and dairy products, largely attributable to contaminated livestock feed (Admasu et al. 2021; Bilandžić et al. 2017; Campagnollo et al. 2016; Chhaya et al. 2023; De Roma et al. 2017; Fallah et al. 2016; Hassan and Kassaify 2014; Li et al. 2017; Matabaro et al. 2017; Zeidan et al. 2022). This study also considered these variables to evaluate their impact on AFM1 contamination.

Recent studies conducted in various countries, including Italy (De Roma et al. 2017) and Serbia (Miocinovic et al. 2017), have revealed high levels of AFM1 in milk, indicating that the presence of this toxin is a global issue. The current research confirmed that all raw milk samples contained AFM1 and demonstrated a seasonal variation in contamination levels, corroborating previous findings. Specifically, in China, AFM1 levels were higher in winter compared to spring, with 1.1% of samples exceeding the European standard limit (Li et al. 2017), similar to the present study's results. In a study conducted by Ismail et al. (2016), in the state of Punjab (Pakistan), 93% of raw milk samples tested positive for AFM1, and 53% exceeded the European Union standard (Ismail et al. 2016). Ismail et al. (2016) reported that in Croatia, 98.4% of raw milk samples were below the European standard limit for AFM1 (Ismail et al. 2016). Research by Campagnollo et al. (2016) indicated that AFM1 contamination was notably higher in winter and spring compared to autumn and summer (Campagnollo et al. 2016). Fallah et al. (2016) stated that in Yazd province (Iran), AFM1 levels in cow, sheep, goat, and camel milk were 46.5%, 21.6%, 20.1%, and 4.03%, respectively. According to Fallah et al. (2016), 15.4% of cow, 11.5% of goat, and 9.15% of sheep milk samples exceeded the Iranian standard (0.050 mg/kg) (Fallah et al. 2016). The elevated AFM1 levels in raw milk during colder seasons are likely due to increased feeding of cows with stored fodder, such as dry hay, concentrates, and silages, which may contain AFB1 (Hashemi 2016). Another limitation is the relatively modest number of samples compared with the overall size of Iran. Although the dataset adequately represents the Tehran supply chain, larger multi‐year surveys would be required for nationwide generalization.

**FIGURE 3:**
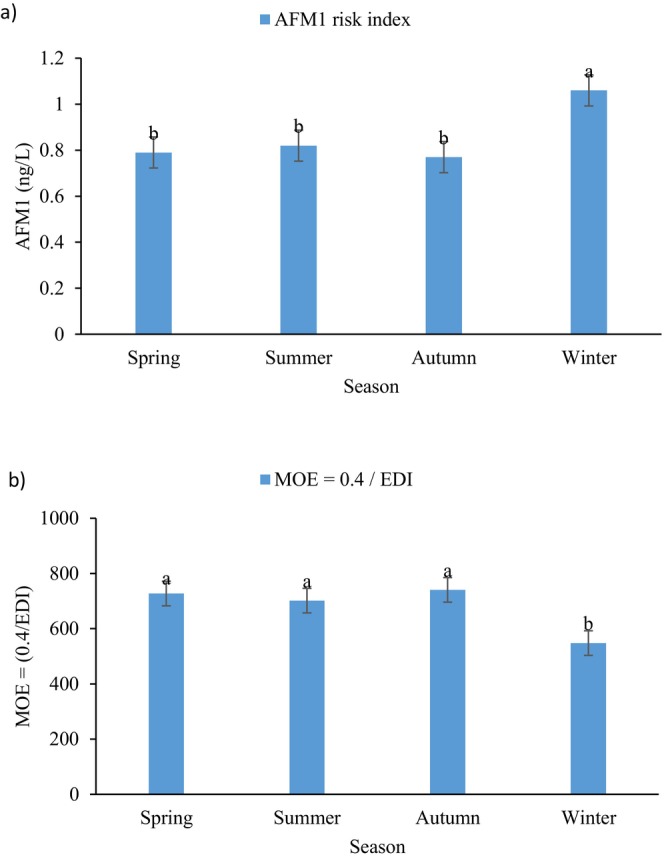
(a) Estimated Daily Intake (EDI) of AFM1 from milk consumption across different seasons. (b) Margin of Exposure (MOE) values for AFM1 intake through milk consumption across seasons. Lowercase letters indicate the statistically difference (*p* ≤ 0.05) between seasons.

The results presented in Figure 3a illustrate the seasonal variation in both the estimated daily intake (EDI) of AFM1 and the corresponding health risk index (HI) associated with milk consumption. Among the four seasons, winter exhibited the highest EDI value (0.73 ng/kg bw/day), corresponding to the lowest MOE (547.9), indicating the greatest potential health concern according to EFSA's benchmark. In contrast, the values recorded for spring, summer, and autumn were lower and remained below the risk threshold, although they still showed statistically significant differences from each other. These findings suggest that seasonal factors, such as variations in livestock feeding practices and storage conditions for animal feed during colder months, may contribute to the elevated AFM1 levels in winter. Overall, the data emphasize the need for enhanced monitoring and preventive strategies, particularly in the winter season, to reduce AFM1 contamination and mitigate its public health impact.

To evaluate the potential health risk associated with AFM1 exposure through milk consumption, the Margin of Exposure (MOE) approach was applied as recommended by EFSA Panel on Contaminants in the Food Chain (CONTAM) et al. (2020). MOE is calculated as the ratio of the benchmark dose lower confidence limit for a 10% response (BMDL10) to the estimated daily intake (EDI) of the compound. For AFM1, a BMDL10 of 0.4 μg/kg bw/day was used, considering its lower carcinogenic potency compared to AFB1. Figure 3b illustrates the MOE associated with milk consumption. Based on our data, the seasonal EDIs of AFM1 were 0.55, 0.57, 0.54, and 0.73 ng/kg bw/day for spring, summer, autumn, and winter, respectively. These values were converted to μg/kg bw/day by dividing by 1000, resulting in MOE values of 727.3, 701.8, 740.7, and 547.9 for the respective seasons. As all MOE values were significantly below the threshold of 10,000, this indicates a potential health concern, particularly for high‐risk groups such as infants and young children. These results emphasize the importance of continuous monitoring and strict regulatory control of AFM1 levels in dairy products.

According to Rahimzadeh Barzoki et al. (2023), EDI values of AFM1 for adults through the consumption of raw milk, pasteurized milk, and UHT milk were reported as 0.09, 0.07, and 0.05 ng/kg body weight per day, respectively. For children, the corresponding EDI values were notably higher, measured at 0.46, 0.37, and 0.27 ng/kg body weight per day, respectively (Rahimzadeh Barzoki et al. 2023). Comparable trends have been observed in other countries such as France, Brazil, and Portugal, where similar levels of aflatoxin exposure through dairy products were documented (Duarte et al. 2013). In Tehran, elevated Estimated Daily Intake (EDI) values related to milk and dairy consumption were reported by Shabansalmani and Movassaghghazani (2023). This rise in contamination during 2020 was likely linked to the presence of mold in livestock feed. However, improvements in feed management practices—such as minimizing extended storage periods—have led to a gradual reduction in aflatoxin contamination across recent years (Shabansalmani and Movassaghghazani 2023). In another investigation, Aghebatbinyeganeh et al. (2024) assessed milk samples from the Iranian provinces of Ilam and Lorestan, reporting hazard index (HI) values below one, indicating negligible health risks (Aghebatbinyeganeh et al. 2024). Similarly, findings from Tabriz showed that adults consuming dairy products such as milk were not exposed to a significant risk of hepatocellular carcinoma (Behtarin and Movassaghghazani 2024). Heshmati et al. (2020) also found no notable public health threat from AFM1 contamination in yogurt samples from Hamedan. These outcomes closely align with the risk assessment results presented in the current study for yogurt (Heshmati et al. 2020).

Table 2 presents the effect of pasteurization on AFM1 contamination levels in milk samples as measured by the HPLC method. The results show that AFM1 concentration was significantly higher in skim milk compared to raw milk (59.40 ± 6.2 vs. 46.20 ± 9.8 ng/L, *p* ≤ 0.05). Additionally, the proportion of samples exceeding the European standard limit of 50 ng/L was greater in skim milk (17.35%) than in raw milk (9.93%). A similar trend was observed when comparing the percentage of samples exceeding the Iranian standard (100 ng/L) and the limits set by the United States (500 ng/L), with skim milk showing higher contamination rates in both cases. These findings suggest that pasteurization does not reduce AFM1 levels; in fact, it may contribute to increased concentrations in the final product, likely due to the removal of fat and the subsequent concentration of AFM1 in the aqueous phase. Consequently, even processed dairy products require strict monitoring and quality control to manage mycotoxin contamination effectively. Zakaria et al. (2019) reported that AFM1 exhibits thermal stability, which means its presence in dairy products is not significantly reduced by common heat treatments such as pasteurization or sterilization. Consequently, conventional food processing methods are ineffective in eliminating this toxin from contaminated products (Zakaria et al. 2019).

**TABLE 2:** Influence of pasteurization on AFM1 (ng/L) levels in milk samples measured using the HPLC method.

Milk	AFM1 content	Above the standards			Minimum	Maximum
		European (> 50 ng/L)	Iranian (> 100 ng/L)	The United States (> 500 ng/L)		
Raw	46.20 ± 4.5^b^	23.87 (9.93%)^a^	4.35 (8.9%)^b^	2.41 (3.5%)^b^	20.0	64.0
Skim	59.40 ± 3.2^a^	18.25 (17.35%)^b^	9.78 (16.11%)^a^	5.65 (8.2%)^a^	30.0	78.0

*Note:* Results reported as MD ± SE. Different lower‐case superscript letters exhibited statistically significant differences (*p* ≤ 0.05).

Table 3 illustrates the impact of processing on AFM1 levels in skim milk samples, as analyzed using the HPLC method. The results clearly demonstrate that AFM1 concentration in powdered skim milk (240.0 ± 27.71 ng/L) was significantly higher than in liquid skim milk (35.0 ± 7.07 ng/L) (*p* ≤ 0.05). The AFM1 levels in powdered samples ranged from 190.0 to 290.0 ng/L, whereas in liquid skim milk, the range was 30 to 40 ng/L. This significant increase suggests that the drying process used to produce milk powder may concentrate AFM1 (Guo et al. 2021), likely due to water removal and the resultant increase in toxin density per volume (Deveci and Sezgin 2006). To better evaluate the effect of the drying process on AFM1 concentration, the reconstituted milk AFM1 level was estimated by dividing the mean value in powdered skim milk (240 ng/L) by the concentration factor (7.5), resulting in an approximate value of 32.0 ng/L. This value is very close to the AFM1 content observed in liquid skim milk samples (35.0 ng/L), indicating that the observed increase in AFM1 concentration in powdered milk is primarily due to water removal during drying, not due to the addition or formation of a new toxin. Therefore, the drying process itself does not appear to affect the actual amount of AFM1 present in the milk, but merely concentrates it by reducing the moisture content. These findings emphasize the importance of evaluating milk processing methods as potential contributors to increased mycotoxin exposure, especially in products with longer shelf lives like powdered milk (Huang et al. 2024). It is important to note that this increase in AFM1 concentration is not due to an increase in the absolute amount of AFM1 but is a consequence of the reduction in milk volume due to fat removal (Einolghozati et al. 2022). Although AFM1 is a metabolite of AFB1 and originates from feed contamination, its chemical nature differs in terms of solubility. Unlike AFB1, which is highly lipophilic, AFM1 is moderately polar and shows a greater affinity for the aqueous phase of milk rather than the fat phase (Ashraf et al. 2024; Yousof et al. 2025). This behavior has been confirmed in previous studies, where skim milk or non‐fat dairy products sometimes showed even higher concentrations of AFM1 due to the reduced volume of fat, which may otherwise dilute the toxin (Aghebatbinyeganeh et al. 2024; Heshmati et al. 2020; Zakaria et al. 2019). Therefore, fat content is not considered a determining factor in AFM1 distribution in milk, which supports the findings observed in the current study. Various studies have shown that pasteurization can reduce the level of AFM1 in milk. For example, in a study conducted by Hassanpour et al. (2019), the aim was to decrease AFM1 in pasteurized milk to a level below the European Codex Alimentarius standard. The study demonstrated that the use of a radioactive granite structure with a low dose of gamma irradiation could effectively reduce AFM1 levels (Hassanpour et al. 2019). In another study published by Mollayusefian et al. (2021), the concentration of AFM1 in raw and pasteurized milk was reported as 57.36 ng/L and 85.39 ng/L, respectively, indicating an increase in AFM1 concentration after pasteurization (Mollayusefian et al. 2021). The use of the MOE (Margin of Exposure) approach is essential for assessing health risks associated with AFM1 in milk. According to EFSA standards, an MOE below 10,000 may indicate potential health concerns. For example, in a study conducted by Kortei et al. (2022), the MOE values for AFM1 in raw milk were reported as 655.7, 1111.1, 2857.1, 5000.00, and 6666.6 (Kortei et al. 2022). Mechanistically, AFM1 is a polar mycotoxin and exhibits stability at the temperatures typically used for pasteurization (72°C for 15 s). Unlike some other toxins, AFM1 does not degrade or volatilize under heat treatment. It is soluble in the aqueous phase of milk, which means that when the fat is removed during pasteurization, the concentration of AFM1 in the remaining liquid phase might actually increase slightly, as observed in our study. This increase is likely due to the concentration effect, where the removal of water during the fat separation process can lead to a higher concentration of AFM1 in the remaining milk (Maggira et al. 2021; Nemati and Khajeali 2025; Zebib et al. 2022).

**TABLE 3:** Effect of spray‐drying on AFM1 (ng/L) concentration in liquid and powdered skim milk.

Milk	AFM1 content	Minimum	Maximum
Skim milk	35.0 ± 7.07^b^	30.0	40.0
Powdered Skim milk	240.0 ± 27.71^a^	190.0	290.0
Reconstituted milk	32.0 ± 5.2^b^	30.0	34.0

*Note:* Results reported as MD ± SE. Different lower‐case superscript letters exhibited statistically significant differences (*p* ≤ 0.05).

Influence of the methods of measurement on AFM1 levels of skim milk samples is shown in Table 4. The results indicate that there was no significant difference between the three evaluated analytical methods in evaluating the AFM1 in milk samples (*p* > 0.05). However, the lowest detection limit is related to HPLC (3 ng/L), followed by ELISA (5 ng/L) and TLC KIT (20 ng/L). These results suggest that immunochromatography is suitable for rapid screening, whereas HPLC remains the reference method for confirmatory testing. Maggira et al. (2021) conducted a comparative study using both ELISA kits and a newly developed HPLC‐Fluorescence method to determine the levels of AFM1 in milk samples. The results of this evaluation indicate that the ELISA method can serve as a rapid and equally reliable alternative to the HPLC method for routine AFM1 analysis in milk. The study highlights the efficiency of ELISA kits in terms of speed and reliability, suggesting their potential use in routine testing environments where quick and accurate results are essential (Maggira et al. 2021). Zhang et al. (2016) reported that the immunochromatographic test strip assay is easy to conduct, rapid, highly sensitive, and specific for the detection of AFM1 residues in milk (Zhang et al. 2016).

**TABLE 4:** Comparison of AFM1 quantification (ng/L) results across immunochromatography, ELISA, and HPLC methods.

Milk	AFM1 content	Detection limit
HPLC	70.50 ± 19.8^a^	3.0
ELISA	72.25 ± 19.1^a^	5.0
TLC KIT	100.0 ± 13.1^a^	20.0

*Note:* Results reported as MD ± SE. Different lower‐case superscript letters exhibited statistically significant differences (*p* ≤ 0.05) between methods.

All analytical methods employed in this study were validated according to international standards. The LOD and LOQ for AFM1 were 3.0 and 10.0 ng/L for HPLC, 5.0 and 15.0 ng/L for ELISA, and 20.0 and 60.0 ng/L for the TLC kit, respectively. Linearity was confirmed over a concentration range of 0–100 ng/L with a correlation coefficient (R^2^) greater than 0.998 for HPLC. The recovery rates for spiked milk samples ranged between 79% and 94%, with RSD values below 10%, indicating acceptable accuracy and precision. Intra‐ and inter‐assay variations for ELISA were also within acceptable limits. No significant matrix effects were observed, confirming the suitability of the methods for AFM1 detection in complex milk matrices. Specificity was ensured by including appropriate positive and negative controls in each analytical run. Overall, the validated methods demonstrated sufficient sensitivity, accuracy, and reproducibility for the quantitative determination of AFM1 in milk samples.

The daily consumption of EDI to assess the carcinogenic effects of AFM1 in milk and dairy consumption was evaluated by all segments of society, and the obtained numbers after placing in the formula as shown in Figure 3a. The milk consumption per capita is 70 kg in Iran. To calculate, the average weight of 60 kg and the mean AFM1 of samples to be 53.64 ng/kg/day were considered. The findings of the health risk assessment revealed that EDI of AFM1 through milk consumption in the Iranian population was 62.58 ng/kg/day. Given that aflatoxins are classified as Group 1 carcinogens by the International Agency for Research on Cancer (IARC), these results highlight the importance of proactive public health policies (Ostry et al. 2017; Ramadan and Al‐Ameri 2022). There is a need for enhanced monitoring and stricter regulation of AFM1 levels in dairy products, particularly during high‐risk seasons (Patyal et al. 2020; Seid and Mama 2019). In addition, improving the quality control and storage conditions of animal feed is essential (Rodríguez‐Blanco et al. 2020), as contaminated feed is the primary source of AFM1 in milk. Educating farmers about proper feed handling practices and raising public awareness about the health risks associated with AFM1 exposure can play a vital role (Yigrem et al. 2025). Promoting consumer access to certified low‐risk dairy products and considering revisions to national regulatory standards to better align with international benchmarks would further help mitigate the long‐term health risks associated with chronic exposure to AFM1 (Massahi et al. 2024). By integrating these strategies into national food safety frameworks, policymakers can reduce population‐wide exposure to AFM1 and enhance public health protection, particularly for vulnerable groups such as children, the elderly, and immunocompromised individuals (Tomasello 2023; Wu et al. 2024). The EDI index for winter was found to be 1.06, suggesting a heightened risk of liver damage and potential liver cancer from milk consumption during this season for Iranian consumers (Bahrami et al. 2016).

It is important to acknowledge that several potential confounding factors may have influenced the observed differences in AFM1 levels across the provinces. Variations in dairy farming practices, such as the use of traditional versus industrial systems (Muaz et al. 2022), differences in feed quality and storage conditions (Muaz et al. 2022), and the types of feed used (e.g., silage, hay, or concentrate), could significantly affect AFM1 contamination levels (Costamagna et al. 2024; Rodríguez‐Blanco et al. 2020). Additionally, economic disparities among regions may influence the ability of farmers to access high‐quality feed and implement proper storage or hygiene practices. These regional differences, along with possible variations in regulatory oversight and farmer education, may have contributed to the variability in AFM1 contamination observed in this study (Alrashedi et al. 2023; Ferrari et al. 2023). Future investigations should aim to control for these variables or include them as part of the analytical framework to better isolate the effect of geographical and seasonal factors on AFM1 levels in milk. Although several studies in Iran have reported AFM1 contamination in milk, our study provides additional novelty by integrating seasonal trends, processing effects, and method comparison within a single framework. Furthermore, by applying the MOE approach for risk assessment, this research aligns with the most updated international recommendations for carcinogenic substances. Together, these contributions extend beyond descriptive monitoring and provide actionable insights for regulatory authorities and public health stakeholders. A limitation of the present study is that monitoring was restricted to one calendar year. Although the seasonal patterns observed are consistent with previous reports, multi‐year datasets are needed to distinguish true climatic effects from short‐term variability.

## 4 Conclusion

This study goes beyond merely updating occurrence data by evaluating the performance of different AFM1 detection methods, assessing the seasonal variation in AFM1 contamination, and investigating the effects of pasteurization and spray drying on AFM1 levels. The immunochromatography method for detecting AFM1 in milk and dairy products demonstrated notable advantages over traditional techniques such as HPLC and ELISA. It proved to be faster, easier to implement, more cost‐effective, and sufficiently reliable for routine screening. Findings from this study confirmed significant seasonal variation in AFM1 contamination, with higher concentrations observed during winter and summer—likely due to differences in feed quality, storage conditions, and animal diet during these periods. Moreover, the research revealed that common heat treatments like pasteurization and spray drying had no significant effect on AFM1 levels, underscoring the thermal stability of this toxin. The seasonal EDI values, combined with MOE estimates well below the 10,000 safety threshold recommended by EFSA, suggest a potential health concern, particularly during winter. Although current AFM1 levels in Iranian milk do not surpass national safety limits (ISIRI), the seasonal trends and associated risks highlight the need for proactive measures. Based on these findings, it is recommended that dairy producers improve the quality control of animal feed, particularly during high‐risk seasons, and adopt better storage practices to minimize fungal growth. Regulatory authorities should strengthen surveillance programs and consider updating national standards to align more closely with international benchmarks. Public health campaigns to raise consumer awareness regarding the risks of AFM1 and the importance of safe feed and milk handling are also essential. These findings are crucial for improving food safety monitoring, regulatory practices, and public health policies related to dairy products in Iran. This study was limited to examining AFM1 contamination in raw milk across Iranian provinces, and there may be a need for further research to include other regions with varying conditions. Additionally, the impact of improving feed management systems and storage conditions on reducing AFM1 levels needs further exploration. Moreover, the long‐term epidemiological effects of chronic AFM1 exposure in vulnerable groups, such as children and the elderly, should be further investigated. Employing newer combined detection methods, along with addressing other factors influencing AFM1 contamination (such as climate and animal management), will be essential for improving the effectiveness of monitoring systems. Future research should explore the development of cost‐effective detoxification strategies for animal feed, the impact of different livestock feeding systems on AFM1 levels, and long‐term epidemiological studies to assess chronic exposure effects in vulnerable populations. Such efforts will support the design of comprehensive risk mitigation plans and contribute to the overall safety of dairy products in Iran.

## Author Contributions

H.R.F.B.: Conceptualization (equal), data curation (equal), formal analysis (equal), investigation (equal), methodology (equal), writing – original draft (equal). M.J.: Methodology (equal), project administration (equal), visualization (equal), writing – original draft (equal), writing – review and editing (equal). Z.A.: Supervision (equal), validation (equal), visualization (equal), writing – original draft (equal), writing – review and editing (equal). B.H.: Methodology (equal), project administration (equal), visualization (equal), writing – original draft (equal), writing – review and editing (equal). R.R.: Writing – review and editing (equal), formal analysis (equal).

## Ethics Statement

The authors have nothing to report.

## Conflicts of Interest

The authors declare no conflicts of interest.

## Supporting information


**Data S1:** fsn371027‐sup‐0001‐supfino.docx.

## Data Availability

Data will be made available on request.
