# Supplementary material for: Monitoring Aflatoxin M1 in Milk From Selected Iranian Provinces Using Different Assays: Seasonal Trends and Processing Effects
Source: Food Sci Nutr. 2025 Sep 26;13(10):e71027. doi: 10.1002/fsn3.71027 (PMC12464443; doi:10.1002/fsn3.71027)
Supplement: Supplementary file 1 — Data S1: fsn371027‐sup‐0001‐supfino.docx. [file FSN3-13-e71027-s001.docx]

| **Table S1.** Average seasonal temperature and relative humidity in 12 Iranian provinces (Spring 2019 – Winter 2020) | | | | | | | | | | | | | |
| --- | --- | --- | --- | --- | --- | --- | --- | --- | --- | --- | --- | --- | --- |
| **Province** | **Season** | **Tehran** | **Esfahan** | **Semnan** | **Qazvin** | **Hamadan** | **Qom** | **Zanjan** | **Kerman** | **Markazi** | **Chaharmahal** | **Mazandaran** | **East Azerbaijan** |
| **Temperature (°C) ± SD** | Spring | 16 ± 2.5 | 18 ± 2.2 | 16 ± 2.3 | 15 ± 2.1 | 14 ± 2.0 | 17 ± 2.1 | 14 ± 2.0 | 18 ± 2.3 | 16 ± 2.1 | 14 ± 2.0 | 17 ± 2.1 | 14 ± 2.0 |
|  | Summer | 33 ± 2.1 | 36 ± 2.0 | 32 ± 1.9 | 31 ± 2.3 | 28 ± 2.4 | 35 ± 2.2 | 30 ± 2.1 | 35 ± 2.1 | 31 ± 2.3 | 27 ± 2.3 | 28 ± 2.2 | 29 ± 2.3 |
|  | Autumn | 21 ± 2.3 | 22 ± 2.1 | 21 ± 2.4 | 20 ± 2.0 | 19 ± 2.3 | 22 ± 2.4 | 19 ± 2.2 | 24 ± 2.4 | 21 ± 2.2 | 19 ± 2.4 | 22 ± 2.1 | 20 ± 2.1 |
|  | Winter | 6 ± 2.7 | 4 ± 2.5 | 3 ± 2.8 | 4 ± 2.6 | 1 ± 2.7 | 6 ± 2.5 | 2 ± 2.8 | 6 ± 2.7 | 3 ± 2.7 | 2 ± 2.6 | 7 ± 2.4 | 1 ± 2.8 |
| **Relative Humidity (%) ± SD** | Spring | 58 ± 5 | 32 ± 4 | 34 ± 4 | 48 ± 5 | 52 ± 5 | 33 ± 4 | 54 ± 6 | 28 ± 4 | 44 ± 5 | 64 ± 7 | 68 ± 6 | 56 ± 5 |
|  | Summer | 27 ± 4 | 22 ± 3 | 18 ± 3 | 33 ± 4 | 38 ± 4 | 23 ± 3 | 38 ± 5 | 18 ± 3 | 33 ± 4 | 43 ± 6 | 73 ± 7 | 42 ± 5 |
|  | Autumn | 48 ± 6 | 34 ± 4 | 28 ± 4 | 44 ± 5 | 48 ± 5 | 36 ± 5 | 46 ± 5 | 32 ± 4 | 43 ± 5 | 58 ± 6 | 63 ± 6 | 52 ± 5 |
|  | Winter | 63 ± 7 | 38 ± 5 | 42 ± 5 | 58 ± 6 | 62 ± 6 | 43 ± 5 | 62 ± 6 | 37 ± 5 | 57 ± 6 | 72 ± 7 | 78 ± 8 | 64 ± 6 |

**Table. S2.** Aflatoxin M1 Concentration Across Dairy Farms and Sampling Seasons

| Farm name | Season | AFM1 (ng/L) | Farm name | Season | AFM1 (ng/L) | Farm name | Season | AFM1 (ng/L) | Farm name | Season | AFM1 (ng/L) | Farm name | Season | AFM1 (ng/L) |
| --- | --- | --- | --- | --- | --- | --- | --- | --- | --- | --- | --- | --- | --- | --- |
| Esfahan Kesht | Spring | 93 | Fazil | Summer | 20 | Zagros | Autumn | 20 | Goldasht | Autumn | 49 | Choopan | Winter | 24 |
| Afzalian | Spring | 20 | FKA | Summer | 20 | Zhal | Autumn | 84 | Choopan | Autumn | 38 | Choopan | Winter | 20 |
| Emdad | Spring | 26 | FKA | Summer | 29 | Zhal | Autumn | 20 | Mahya | Winter | 20 | Ne'mat Bakhsh | Winter | 85 |
| Shad Ice Cream | Spring | 23 | Ghiam | Summer | 76 | Pajen | Autumn | 53 | Emdad | Winter | 20 | Ne'mat Bakhsh | Winter | 20 |
| Shad Ice Cream | Spring | 20 | Ghiam | Summer | 20 | Pajen | Autumn | 36 | Nasr | Winter | 78 | Noor Afarin | Winter | 20 |
| Pajen | Spring | 20 | Ghiam | Summer | 31 | Pajen | Autumn | 20 | Bistoon | Winter | 71 | Goldasht | Winter | 74 |
| Pajen | Spring | 20 | Zagros | Summer | 22 | Pakban | Autumn | 20 | Pajen | Winter | 48 | Laban Pak | Winter | 20 |
| Pahlevan-Nejad | Spring | 20 | Zagros | Summer | 20 | Emdad | Autumn | 20 | Pajen | Winter | 20 | Esfahan Kesht | Winter | 150 |
| Khorram Dareh | Spring | 26 | Zarrin Hiv | Summer | 20 | Halib | Autumn | 20 | Pakban | Winter | 34 | Esfahan Kesht | Winter | 100 |
| Domino | Spring | 80 | Alian | Summer | 64 | Adabi | Autumn | 55 | Najafian | Winter | 150 | Pahlevan-Nejad | Winter | 81 |
| Dehkadeh | Spring | 81 | Fajr Esfahan | Summer | 20 | Afradasht | Autumn | 61 | Najafian | Winter | 69 | Shad Ice Cream | Winter | 45 |
| Dehkadeh | Spring | 20 | Fajr Esfahan | Summer | 20 | Afradasht | Autumn | 26 | Najafian | Winter | 134 | Shad Ice Cream | Winter | 64 |
| Dehkadeh | Spring | 20 | Fazil | Summer | 93 | Afzalian | Autumn | 57 | Bistoon | Winter | 132 | Shad Ice Cream | Winter | 150 |
| Zhal | Spring | 20 | Goldasht | Summer | 20 | Emdad | Autumn | 82 | Zhal | Winter | 146 | Shad Ice Cream | Winter | 82 |
| Safinia | Spring | 71 | Laban Pak | Summer | 55 | Semnan Coop | Autumn | 41 | Zhal | Winter | 35 | Dasht Gol | Winter | 43 |
| Ali Naqian | Spring | 55 | Maxal | Summer | 52 | Semnan Coop | Autumn | 40 | Alian | Winter | 57 | Afzalian | Winter | 20 |
| Ali Naqian | Spring | 140 | Mahnoosh | Summer | 24 | Semnan Coop | Autumn | 20 | Fazil | Winter | 30 | Dehkadeh | Winter | 80 |
| Ali Naqian | Spring | 150 | Kalleh | Summer | 100 | Semnan Coop | Autumn | 26 | Fazil | Winter | 20 | Dehkadeh | Winter | 20 |
| Ali Naqian | Spring | 75 | Malard | Summer | 36 | Taliseh | Autumn | 36 | FKA | Winter | 51 | Maxal | Winter | 150 |
| Fajr Esfahan | Spring | 43 | Kalleh | Summer | 110 | Taliseh | Autumn | 20 | FKA | Winter | 20 | Najafian | Winter | 150 |
| Fajr Esfahan | Spring | 20 | Vadi Sabz | Summer | 45 | Khorram Dareh | Autumn | 34 | FKA | Winter | 20 | Najafian | Winter | 150 |
| Fazil | Spring | 32 | Zare | Summer | 67 | Shad Ice Cream | Autumn | 94 | FKA | Winter | 20 | Najafian | Winter | 150 |
| Fazil | Spring | 20 | NematBakhsh | Summer | 47 | Shad Ice Cream | Autumn | 63 | Ghiam | Winter | 120 | Najafian | Winter | 150 |
| FKA | Spring | 99 | NematBakhsh | Summer | 21 | Shad Ice Cream | Autumn | 71 | Malayer | Winter | 104 | Nissani | Winter | 105 |
| FKA | Spring | 20 | Vadi Sabz | Summer | 43 | Pahlevan-Nejad | Autumn | 20 | Malayer | Winter | 110 | Afradasht | Winter | 20 |
| Ghiam | Spring | 57 | Kalleh | Summer | 100 | Najafian | Autumn | 20 | Malayer | Winter | 41 | Afradasht | Winter | 20 |
| Goldasht | Spring | 27 | Taliseh | Summer | 140 | Najafian | Autumn | 20 | Khojand | Winter | 76 | Sarhadi | Winter | 46 |
| Goldasht | Spring | 20 | Taliseh | Summer | 25 | Najafian | Autumn | 20 | Domino | Winter | 23 | Sarhadi | Winter | 84 |
| Choopan | Spring | 20 | Taliseh | Summer | 50 | Najafian | Autumn | 33 | Rabta | Winter | 39 | Sarhadi | Winter | 20 |
| Laban Pak | Spring | 45 | Dasht Gol | Summer | 150 | Mahya | Autumn | 57 | Rabta | Winter | 20 | Zare | Winter | 57 |
| Laban Qom | Spring | 24 | Dehkadeh | Summer | 33 | Maxal | Autumn | 32 | Rabta | Winter | 20 | Zare | Winter | 44 |
| Laban Qom | Spring | 111 | Dehkadeh | Summer | 20 | Maxal | Autumn | 50 | Najafian | Winter | 110 | Nasr | Winter | 34 |
| Laban Qom | Spring | 67 | Emdad | Summer | 20 | Najafian | Autumn | 100 | Najafian | Winter | 80 | Safinia | Winter | 20 |
| Mahya | Spring | 20 | Emdad | Summer | 20 | Najafian | Autumn | 20 | Najafian | Winter | 130 | Shad Ice Cream | Winter | 108 |
| Mahnoosh | Spring | 42 | Emdad | Summer | 42 | Ali Naqian | Autumn | 150 | Najafian | Winter | 20 | Esfahan Kesht | Winter | 20 |
| Nasr | Spring | 26 | Halib | Summer | 28 | Fajr Esfahan | Autumn | 20 | Najafian | Winter | 36 | Semnan Coop | Winter | 150 |
| Noor Afarin | Spring | 20 | Malayer | Summer | 20 | Khojand | Autumn | 92 | Najafian | Winter | 20 | Esfahan Kesht | Winter | 150 |
| Nissani | Spring | 37 | Malayer | Summer | 69 | Dasht Gol | Autumn | 65 | Najafian | Winter | 150 | Ali Naqian | Winter | 26 |
| Zare | Spring | 49 | Pajen | Summer | 51 | Domino | Autumn | 67 | Kalber | Winter | 24 | Ali Naqian | Winter | 20 |
| Zare | Spring | 20 | Adabi | Summer | 78 | Fazil | Autumn | 75 | Kalber | Winter | 20 | Ali Naqian | Winter | 20 |
| Malard | Spring | 150 | Pajen | Autumn | 92 | FKA | Autumn | 64 | Adabi | Winter | 64 | Fajr Esfahan | Winter | 48 |
| Khorram Dareh | Summer | 146 | Domino | Autumn | 51 | FKA | Autumn | 45 | Emdad | Winter | 35 | Fajr Esfahan | Winter | 20 |
| Esfahan Kesht | Summer | 23 | Rabta | Autumn | 50 | FKA | Autumn | 20 | Emdad | Winter | 20 | Zagros | Winter | 20 |
| Shad Ice Cream | Summer | 32 | Rabta | Autumn | 20 | Ghiam | Autumn | 69 | Zarrin | Winter | 28 | Total | 227 | |
| Shad Ice Cream | Summer | 70 | Zagros | Autumn | 32 | Choopan | Autumn | 73 | Nissani | Winter | 106 |  |  |  |
| Pahlevan-Nejad | Summer | 20 | Bistoon | Autumn | 20 | Bistoon | Autumn | 20 | Zagros | Winter | 63 |  |  |  |

**Method validation procedures are provided in the Supplementary Materials**

All analytical methods, including immunochromatography, ELISA, and HPLC, were validated in accordance with international guidelines. Calibration was performed using certified AFM1 standard solutions for each method. For immunochromatographic tests, positive and negative controls were included in each run to ensure specificity and consistency. The ELISA method was evaluated for intra- and inter-assay precision using milk samples spiked at three levels (25, 50, and 100 ng/L). Recovery and coefficient of variation (CV) were calculated across three independent sessions with different operators and reagent batches. LOD and LOQ were determined based on the standard deviation of the blank and the slope of the calibration curve. Matrix effects were assessed by comparing solvent-based and matrix-matched standards. HPLC validation included linearity (R² > 0.998), specificity, recovery, and sensitivity. Spiked samples at 0–100 ng/L were analyzed in quintuplicate. LOD and LOQ were established using signal-to-noise ratios of 3 and 10, respectively. Recovery rates ranged between 79% and 94%, with RSD <10%. Matrix effects were negligible, indicating the method’s suitability for accurate AFM1 quantification in milk samples.
